# *Solanum aculeatissimum* and *Solanum torvum* chloroplast genome sequences: a comparative analysis with other Solanum chloroplast genomes

**DOI:** 10.1186/s12864-024-10190-9

**Published:** 2024-04-26

**Authors:** Longhao Zhang, Chengqi Yi, Xin Xia, Zheng Jiang, Lihui Du, Shixin Yang, Xu Yang

**Affiliations:** College of Horticulture and Landscape Architecture, Yangzhou University, 225009 Yangzhou, China

**Keywords:** *Solanum aculeatissimum*, *Solanum torvum*, Chloroplast genome, Phylogenetic analysis

## Abstract

**Background:**

*Solanum aculeatissimum and Solanum torvum* belong to the *Solanum* species, and they are essential plants known for their high resistance to diseases and adverse conditions. They are frequently used as rootstocks for grafting and are often crossbred with other *Solanum* species to leverage their resistance traits. However, the phylogenetic relationship between *S. aculeatissimum* and *S. torvum* within the *Solanum* genus remains unclear. Therefore, this paper aims to sequence the complete chloroplast genomes of *S. aculeatissimum* and *S. torvum* and analyze them in comparison with 29 other previously published chloroplast genomes of *Solanum* species.

**Results:**

We observed that the chloroplast genomes of *S. aculeatissimum* and *S. torvum* possess typical tetrameric structures, consisting of one Large Single Copy (LSC) region, two reverse-symmetric Inverted Repeats (IRs), and one Small Single Copy (SSC) region. The total length of these chloroplast genomes ranged from 154,942 to 156,004 bp, with minimal variation. The highest GC content was found in the IR region, while the lowest was in the SSC region. Regarding gene content, the total number of chloroplast genes and CDS genes remained relatively consistent, ranging from 128 to 134 and 83 to 91, respectively. Nevertheless, there was notable variability in the number of tRNA genes and rRNAs. Relative synonymous codon usage (RSCU) analysis revealed that both *S. aculeatissimum* and *S. torvum* preferred codons that utilized A and U bases. Analysis of the IR boundary regions indicated that contraction and expansion primarily occurred at the junction between SSC and IR regions. Nucleotide polymorphism analysis and structural variation analysis demonstrated that chloroplast variation in *Solanum* species mainly occurred in the LSC and SSC regions. Repeat sequence analysis revealed that A/T was the most frequent base pair in simple repeat sequences (SSR), while Palindromic and Forward repeats were more common in long sequence repeats (LSR), with Reverse and Complement repeats being less frequent. Phylogenetic analysis indicated that *S. aculeatissimum* and *S. torvum* belonged to the same meristem and were more closely related to Cultivated Eggplant.

**Conclusion:**

These findings enhance our comprehension of chloroplast genomes within the *Solanum* genus, offering valuable insights for plant classification, evolutionary studies, and potential molecular markers for species identification.

**Supplementary Information:**

The online version contains supplementary material available at 10.1186/s12864-024-10190-9.

## Background

The *Solanaceae* family holds a pivotal position in the realm of vegetables, not only for its extensive population but also for the substantial economic value of *Solanaceae* crops [1]. Comprising over 90 genera, the *Solanaceae* family extends its influence beyond the realm of food, playing a crucial role in industry and scientific exploration [2]. *Solanum*, as a prominent component of the *Solanaceae* family, boasts a vast array of species distributed far and wide. Yet, the sheer size and monophyletic nature of *Solanum* plants often present challenges in their classification and analysis [3, 4]. Moreover, the propensity of certain *Solanaceae* species to hybridize with one another blurs the lines of strict reproductive isolation within the family [5, 6]. While this phenomenon fosters crossbreeding and germplasm resource innovation, it simultaneously complicates species identification, delineation of kinship, and taxonomic categorization [6, 7].

Chloroplasts, a type of plastid commonly found in plants, harbor their own complete genome and serve as crucial organelles with autonomous genetic information within plant cells. Research has consistently demonstrated the highly conserved nature of chloroplast genome structures in the majority of flowering plants. Due to the gradual evolutionary pace of chloroplast genomes, they have been extensively employed in plant classification and molecular evolutionary studies. The use of whole chloroplast genomes for species identification and phylogenetic investigations represents a burgeoning trend in the field of plant phylogenetic biology, gaining increasing attention and recognition from scholars [8, 9].

*Solanum aculeatissimum* and *Solanum torvum*, two wild relatives of the eggplant, exhibit remarkable tenacity and resilience, particularly in their resistance to soil-borne diseases like wilt and yellow wilt [10]. Additionally, they possess certain medicinal and edible qualities. Consequently, the exploration of their phylogenetic evolutionary relationships carries significant implications for enhancing disease resistance, stress tolerance, and fruit quality in the broader *Solanum* genus. In pursuit of this objective, our study delves into the chloroplast genomes of 31 *Solanum* plants, comparing their chloroplast structures, constructing a genus-level phylogeny, and dissecting the relationships among *Solanum* species. The overarching goal of this research is to furnish a reference point for the cultivation of intermediate hybrids within the *Solanum* genus.

## Materials and methods

### DNA extraction and sequencing

The materials used in this study were sourced from 222 laboratories within the College of Horticulture and Landscape Architecture (32°23′N, 119°24′E), Yangzhou University. Two solanaceous species, *S. aculeatissimum* and *S. torvum*, were selected for chloroplast genome sequencing. Healthy young leaves of *S. aculeatissimum* and *S. torvum* were collected from the experimental fields at Yangzhou University. The collected leaves were immediately placed into liquid nitrogen and stored at -80 °C. Genomic DNA extraction method using kit extraction (Plant DNA Isolation Mini Kit-BOX2 Vazyme Cat.DC104-01). The extracted DNA was then randomly sheared into smaller fragments using an Ultrasound Covaris instrument, resulting in a series of DNA fragments.

Subsequently, the fragmented DNA underwent purification, end repair, and 3’ end A-tailing. The quality of the DNA was assessed through agarose gel electrophoresis and spectrophotometry. Fragment size selection was performed via agarose gel electrophoresis, followed by PCR amplification to generate a sequencing library. The library underwent initial quality assessment, and once deemed qualified, it was subjected to sequencing using Illumina HiSeq platform technology. Genomic DNA quality and quantity were evaluated using the Nanodrop detection method. The experimental procedures adhered to the standard protocol provided by Nanjing Genepioneer Biotechnologies, Inc. (Nanjing, China), encompassing sample quality testing, library construction, library quality assessment, and library sequencing. The sequencing was conducted in a paired-end (PE150) format, with the sequencing data presented in Table S1. Using fastp v0.23.4 (https://github.com/opengene/fastp)Th The software filters the raw data according to the following filtering criteria: 1、Remove sequencing connectors and primer sequences from Reads. 2、Filter out reads with average quality values less than Q5. 3、Filter out N(empty base)reads greater than 5.The high-quality Reads obtained after the above series of quality control are called Clean Data.

### Chloroplast assembly and annotation

Chloroplast genome assembly for *S. aculeatissimum* and *S. torvum* utilized clean data and was conducted using GetOrganelle v1.7.2 [11]. The reference sequence used in the assembly can be retrieved from NCBI (https://www.ncbi.nlm.nih.gov/nuccore/MN218080.1/)To confirm the ring-like structure of the assembled data, we employed the visualization software Bandage v0.8.1 [12]. Sequences obtained from the assembly were subjected to BLAST analysis on NCBI (https://blast.ncbi.nlm.nih.gov/Blast.cgi), and the sequence with the highest similarity was selected as a reference for prediction and annotation (https://www.ncbi.nlm.nih.gov/nuccore/MN218087.1/, https://www.ncbi.nlm.nih.gov/nuccore/NC_061388.1/), using the default parameters of CPGAVAS2 (http://47.96.249.172:16019/analyzer/annotate) [13]. The data generated in the previous steps were manually refined using Apollo v1.11.8 [14] to produce the final annotated file. The annotated GenBank (gbf) files were used to visualize the chloroplast genome structures through an online tool available at this URL (https://irscope.shinyapps.io/Chloroplot/). Additionally, tRNAs were analyzed using tRNAscan-SE v2.0 software [15]. Relative synonymous codon usage (RSCU) was detected using CodonW v 1.4.2 [16].

### Repeat sequence identification

Repeat sequences within the chloroplast genomes of the 31 *Solanum* species were analyzed for Simple Sequence Repeats (SSR) using the online tool MISA (https://webblast.ipk-gatersleben.de/misa/index.php?action=1) [17]. Mononucleotide, dinucleotide, trinucleotide, tetranucleotide, pentanucleotide, andhexanucleotide were set to 10, 5, 4, 3, 3, and 3. To detect scattered repeat sequences, we employed the online software REPuter (https://bibiserv.cebitec.uni-bielefeld.de/reputer/) [18]. Additionally, Tandem Repeat sequences were identified using TandemRepeatFinder (TRF) (https://tandem.bu.edu/trf/trf.html), with default parameters.

### Comparative genome analysis

Comparative genome analysis encompassed the examination of 31 chloroplast genomes from *Solanum* species. The expansion and contraction of the Inverted Repeat (IR) regions between Large Single Copy (LSC), Small Single Copy (SSC), and IR were assessed using IRScope (https://irscope.shinyapps.io/irapp/) [19]. Multiple sequence comparison of chloroplast genomes was conducted using MAFFT v7.487 [20]. The results of this comparison were then input into DnaSP v.16.3 [21] to calculate nucleotide diversity (Pi). The step size was set to 200 bp, and the sliding window length was set to 600 bp. Additionally, to corroborate the analysis results, chloroplast variation was assessed using mVISTA(https://genome.lbl.gov/vista/mvista/submit.shtml) [22],upload the prepared sequence files of *S. aculeatissimum* and *S. torvum* to this website and select the Shuffle-LAGAN mode in the Alignment programme. Finally, manual counting was employed to determine the number of deletions in the 31 chloroplast genes of *Solanum* species.

### Phylogenetic analysis

For phylogenetic analysis, a tree was constructed using chloroplast genomes from 31 species of *Solanum* and three species of Tobacco as reference. Common genes were extracted using PhyloSuite v1.2.3 [23] from all sequences before tree construction. The extracted common genes underwent multiple sequence comparison using MAFFT v7.487 [20]. Subsequently, Using phyloSuite to link up the genes after comparision,the linked common genes were optimized using Gblocks v0.91b [24]. The best model for phylogenetic analysis was determined using IQTREE v1.6.8 [25], The best model for phylogenetic analysis was determined using IQTREE v1.6.8, The optimal model for this study, determined through IQTREE, is TVM + F + I + G4 of BIC. The divergence time of the reference species was obtained by querying the website (http://timetree.org/). The substitution model selected was GTR, the site heterogeneity model was set to Gamma, and the clock type chosen was Uncorrelated Relaxed Clock. The length of the chain was set to 100 million, while all other settings were maintained at their default parameters.

The generated files were used to estimate species divergence times using Beast v1.8.4 [26], and finally the generated files were used to build a tree using Figtree v1.4.4 [27].

## Results

### Genome characteristics

**Fig. 1:**
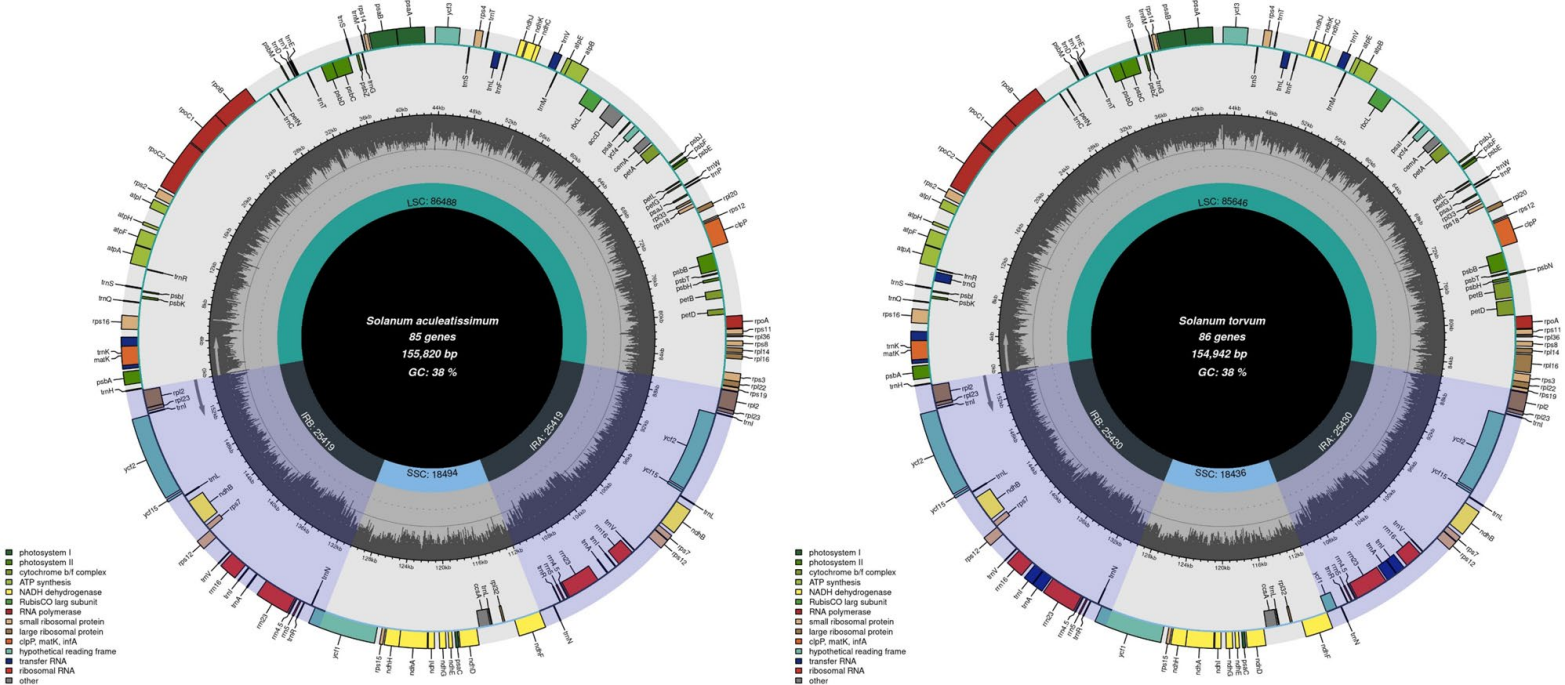
Chloroplast genome map of *Solanum aculeatissimum* and *Solanum torvum*

By sequencing, we obtained the chloroplast whole genomes of *S. aculeatissimum* and *S. torvum*, which were 155,820 bp and 154,942 bp in length, respectively. Simultaneously, we downloaded the chloroplast whole genomes of 29 other *Solanum* species from the official NCBI website and analyzed them. The chloroplast genomes of *S. aculeatissimum* and *S. torvum* exhibit typical tetrameric structures, consisting of one LSC (Large Single Copy), two reverse-symmetric IRs (Inverted Repeats), and one SSC (Small Single Copy) region (Fig. 1). The total length of the chloroplast genomes in these 31 *Solanum* species ranged from 154,942 to 156,004 bp. Specifically, the LSC region ranged from 85,646 to 86,667 bp, the IR region ranged from 25,417 to 25,639 bp, and the SSC region ranged from 18,347 to 18,609 bp (Table 1). While the chloroplast genome of *S. torvum* was the shortest among the newly sequenced species, in general, the size of chloroplast genomes in *Solanum* showed limited variation and remained relatively conservative.

Regarding the distribution of GC content, all *Solanum* species exhibited the highest GC content in the IR region and the lowest in the SSC region. For instance, the GC contents in the LSC, IR, and SSC regions of *S. aculeatissimum* were 35.9%, 43.09%, and 31.9%, respectively, while in *S. torvum*, they were 35.92%, 43.08%, and 32.04%, respectively. The total number of chloroplast genes and the total number of CDS genes remained relatively constant, ranging from 128 to 134 and 83 to 91, respectively. However, there was significant variability in the number of tRNA genes and rRNAs. The minimum number of tRNAs was observed in *S. aculeatissimum*, with only 32, whereas *S. commersonii* had the maximum number of tRNAs at 39. In terms of rRNAs, all 31 Solanum species had 8. Specifically, in *S. aculeatissimum*, 11 genes contained introns, while in *S. torvum*, 12 genes had introns. Among these genes with introns, six were tRNAs (tRNA-UUU, tRNA-CGA, tRNA-UUC, tRNA-UAA, tRNA-UACand tRNA-UGC), and six were other intronic genes (Tables S2 and S3).

**Table 1:** Sample information and summary of 31 chloroplast genome characteristics of solanum species

Species	Plastome		LSC		IR		SSC		Number of all genes	Number of CDS genes	Number of tRNA genes	Number of rRNA genes	Data sources
	Size (bp)	GC (%)	Size (bp)	GC (%)	Size (bp)	GC (%)	Size (bp)	GC (%)					
*S.acaule*	155,570	37.84	86,020	35.95	25,593	43.09	18,364	32.08	132	87	37	8	MK036506
*S.aculeatissimum*	155,820	37.77	86,466	35.9	25,419	43.09	18,494	31.9	128	84	36	8	OR381845
*S.aethiopicum*	155,606	37.7	86,164	35.78	25,440	43.06	18,563	31.95	132	90	34	8	MN218076
*S.anguivi*	155,753	37.66	86,261	35.75	25,442	43.02	18,609	31.88	132	90	34	8	MN218088
*S.berthaultii*	155,533	37.88	85,975	36.01	25,593	43.1	18,372	32.1	132	91	33	8	KY419708
*S.brevicaule*	155,531	37.87	85,981	35.99	25,599	43.08	18,354	32.12	132	91	33	8	MK036507
*S.bulbocastanum*	155,371	37.88	85,785	36.02	25,588	43.08	18,381	32.13	134	89	37	8	DQ347958
*S.campylacanthum*	155,017	37.72	86,667	35.87	25,422	43.06	18,506	31.91	134	90	36	8	NC_039609.1
*S.cardiophyllum*	155,570	37.9	85,983	36.04	25,596	43.08	18,395	32.18	132	87	37	8	MK690622
*S.chacoense*	155,532	37.89	85,972	36.02	25,592	43.09	18,376	32.15	132	87	37	8	MF471371
*S.commersonii*	155,525	37.88	86,013	36	25,573	43.12	18,366	32.09	133	86	39	8	NC028069
*S.dasyphyllum*	155,716	37.63	86,314	35.68	25,433	43.03	18,536	31.89	132	87	37	8	MH283716
*S.demissum*	155,558	37.87	85,999	36	25,593	43.1	18,373	32.1	132	87	37	8	MK036508
*S.hougasii*	155,549	37.87	85,990	36	25,593	43.1	18,373	32.09	132	87	37	8	MF471372
*S.incanum*	155,657	37.7	86,276	35.78	25,421	43.06	18,539	31.91	134	90	36	8	NC039605
*S.lichtensteinii*	155,574	37.71	86,217	35.79	25,421	43.06	18,515	31.93	134	90	36	8	NC_039598.1
*S.linnaeanum*	155,574	37.7	86,219	35.78	25,423	43.06	18,509	31.93	134	90	36	8	NC_039600.1
*S.lycopersicum*	155,461	37.86	85,876	35.99	25,611	43.09	18,363	32.04	134	89	37	8	NC_007898.3
*S.macrocarpon*	156,004	37.61	86,542	35.65	25,462	43.01	18,538	31.92	132	86	38	8	MN218081.1
*S.melongenaon*	155,581	37.71	86,194	35.8	25,443	43.05	18,501	31.95	132	86	38	8	MN218080
*S.peruvianum*	155,561	37.84	85,906	35.98	25,639	43.06	18,377	32.02	128	83	37	8	KP117026
*S.pimpinellifolium*	155,442	37.88	85,856	36.01	25,612	43.09	18,364	32.08	128	83	37	8	NC026882
*S.polhillii*	155,422	37.67	86,044	35.72	25,417	43.08	18,546	31.9	134	90	36	8	NC_039414.1
*S.rostratum*	155,559	37.76	86,281	35.87	25,418	43.07	18,442	31.91	130	85	37	8	NC_057245.1
*S.sisymbriifolium*	155,771	37.76	86,404	35.85	25,421	43.08	18,527	32.06	132	86	38	8	MN218090
*S.supinum*	155,769	37.68	86,362	35.74	25,429	43.06	18,549	32	134	90	36	8	NC_039601.1
*S.torvum*	154,942	37.81	85,646	35.92	25,430	43.08	18,436	32.04	130	85	37	8	OR381846
*S.tuberosum*	155,296	37.88	85,737	36.01	25,593	43.1	18,375	32.09	133	88	37	8	NC_008096.2
*S.umtuma*	155,541	37.7	86,173	35.79	25,427	43.04	18,514	31.94	134	90	36	8	NC_039413.1
*S.verrucosum*	155,479	37.88	85,876	36	25,593	43.1	18,347	32.1	130	85	37	8	NC_041632.1
*S.wrightii*	155,506	37.68	86,231	35.77	25,423	43.07	18,429	31.78	132	86	38	8	MN218084

### Relative synonymous codon usage

Based on the coding sequence (CDS), we estimated the codon usage frequency, specifically the relative synonymous codon usage (RSCU), for *S. aculeatissimum* and *S. torvum*. In total, there are 26,247 codons present in all protein coding genes of *S. aculeatissimum*. Among these codons, leucine was the most abundant amino acid, accounting for 10.64% of the total (2,792 codons). Isoleucine was the second most abundant at 8.38%, while cysteine was relatively rare, constituting only 1.15% of the codons. This observation aligns with previous findings indicating that leucine and isoleucine are the most common amino acids in angiosperms [28, 29]. Additionally, tryptophan is encoded by a single codon (UGG), implying no codon bias. The RSCU values for nearly all A/U termination codons exceeded 1, while those for C/G termination codons were below 1 (Table S4).

In the case of *S. torvum*, leucine was the most abundant amino acid in its chloroplast, accounting for 10.74% of the total (2,827 codons). Isoleucine was the second most common, making up 8.43% (2,219 codons), while cysteine was the least abundant at 1.14% (299 codons), similar to *S. aculeatissimum*. Similarly, tryptophan exhibited no codon bias. The RSCU analysis revealed that out of 18,430 codons with RSCU values greater than 1, 17,237 ended with A and U, indicating a preference for A and U bases in the third codon position. Among all codons in *S. torvum*, AUG had the highest RSCU value, followed by UUA and GCU, while UUG had the lowest (Table S5).

### IR contraction and expansion

Through the analysis of fundamental features in the chloroplast genomes of *Solanum* species, it has been revealed that there exists an approximately 1000 bp gap in these genomes. The size variations in plant chloroplast genomes primarily result from the expansion and contraction of the IR and SSC regions [30, 31]. Therefore, it is highly likely that the chloroplasts of *Solanum* species undergo both contraction and expansion at the IR boundary. In this study, we analyzed the chloroplast genomes of.

**Fig. 2:**
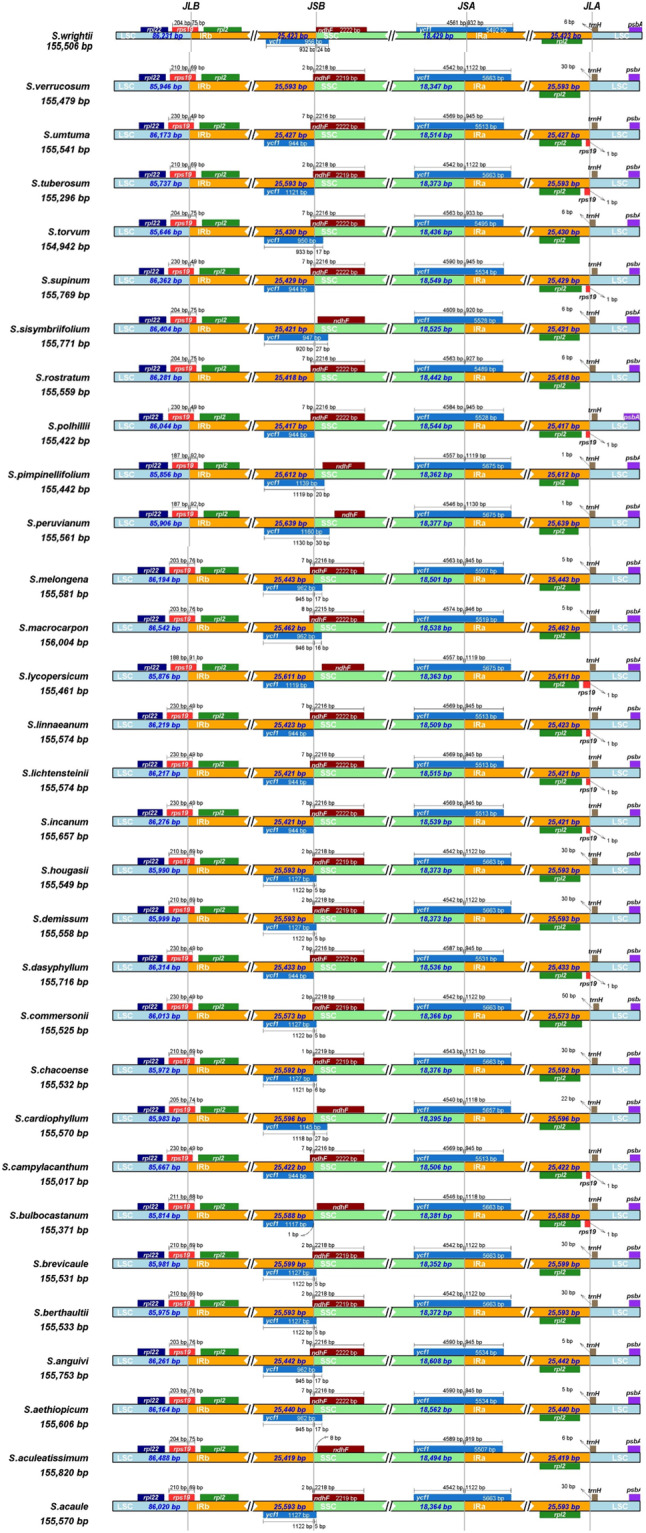
Comparison of the boundaries of the LSC, SSC and IR regions. JLB: junction between LSC and IRb; JSB: junction between SSC and IRb; JSA: junction between. SSC and IRa; JLA: junction between LSC and IRa

31 *Solanum* species to investigate the expansion and contraction of the IR boundary (Fig. 2).

The results indicated that rps19 was consistently present at the IRb-LSC boundary, albeit with varying degrees of shifts among different species. The situation at the IRb-SSC boundary was more intricate, with some species harboring both *ycf1* and *ndhF* genes at this boundary, while others had only *ndhF* without *ycf1*. Notably, species such as *S. aculeatissimum*, *S. rostratum*, and *S. verrucosum* lacked *ycf1* at this boundary altogether, and there was an outlier group of species, including *S. aculeatissimum* and *S. verrucosum*, that had no genes at this boundary. The situation at the IRa-SSC boundary was simpler, with only one gene, *ycf1*, present. The pattern at the IRa-LSC boundary closely mirrored that of the IRb-SSC boundary, characterized by the alternation of *rps19* and *trnH* within this region.

### Comparative analysis of genome structure

**Fig. 3:**
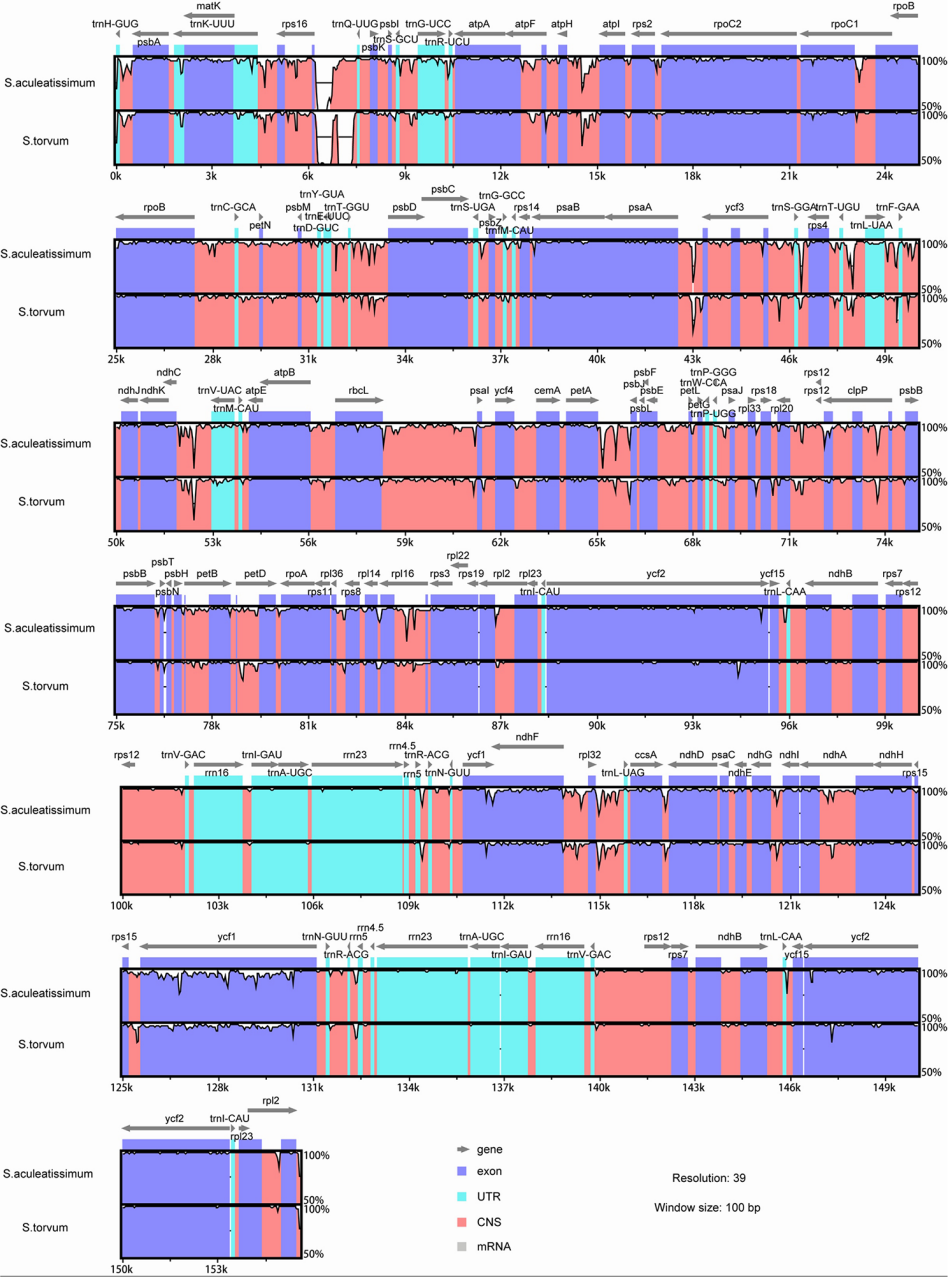
Comparison of fve chloroplast genomes using mVISTA by taking *Solanum melongenaon* sequence as a reference. The lower left corner is the color coding of gene function, grey arrows indicate the orientation of genes, red bars represent conserved non-coding sequences, purple bars represent exons, and blue bars represent introns. The y-axis represents the percentage identity (shown: 50–100%)

The chloroplast genomes of *S. aculeatissimum* and *S. torvum* were compared using mVISTA, with *S. melongena* as the reference (Fig. 3). The analysis revealed that the primary regions of variation in the chloroplast genomes of *S. aculeatissimum* and *S. torvum* were the LSC and SSC regions, aligning with findings observed in other plant species [32]. Notably, a gap was even identified within the LSC region, indicating that the similarity between the two genomes in this specific location was less than 50%.

### Simple sequence repeat and long repeats analysis

**Fig. 4:**
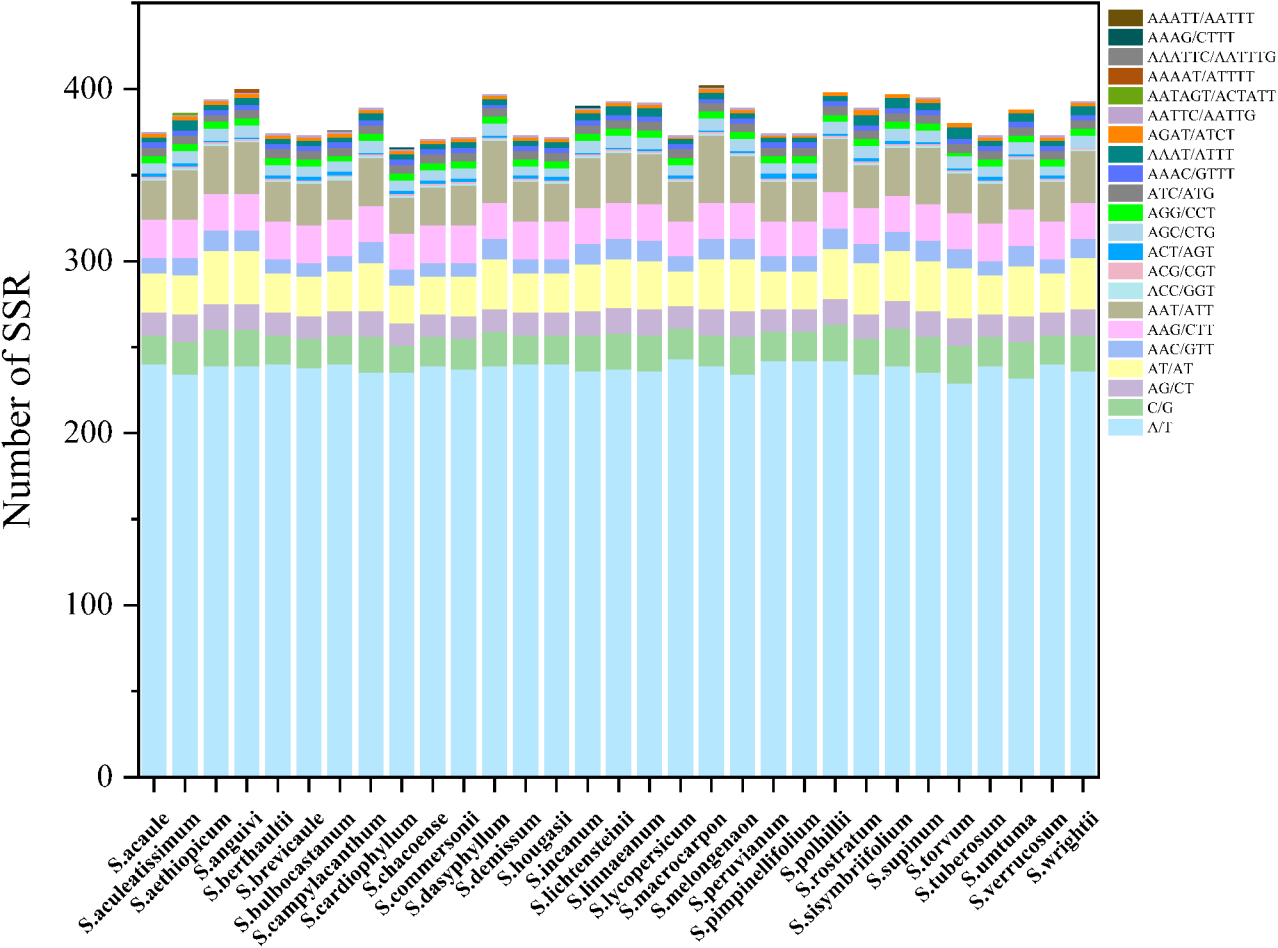
Analysis of simple sequence repeats (SSRs) in the *Solanum* chloroplast genomes

Simple repeat sequences (SSRs) are among the more common molecular markers, typically consisting of tandem repeats of 1–6 base pairs of DNA [33–35]. In our analysis of 31 *Solanum* species, a total of 22 types of SSRs were detected using MISA (Fig. 4 and Table S6). The most prevalent type of SSR in *Solanum* was A/T, accounting for approximately 60% of the total number of SSRs. As the number of base pairs increased, the quantity of other SSR types decreased.

While the majority of *Solanum* plants shared common types of SSRs, there were unique SSRs identified in the chloroplast genomes of individual species. For instance, *S. aculeatissimum* exhibited the AATAGT/ACTATT SSR, *S. bulbocastanum* had AAATTC/AATTTG, *S. macrocarpon* displayed AAATT/AATTTG, *S. anguivi* showed AAAATT/AATTT. These unique SSRs can serve as valuable molecular markers for species identification.

**Fig. 5:**
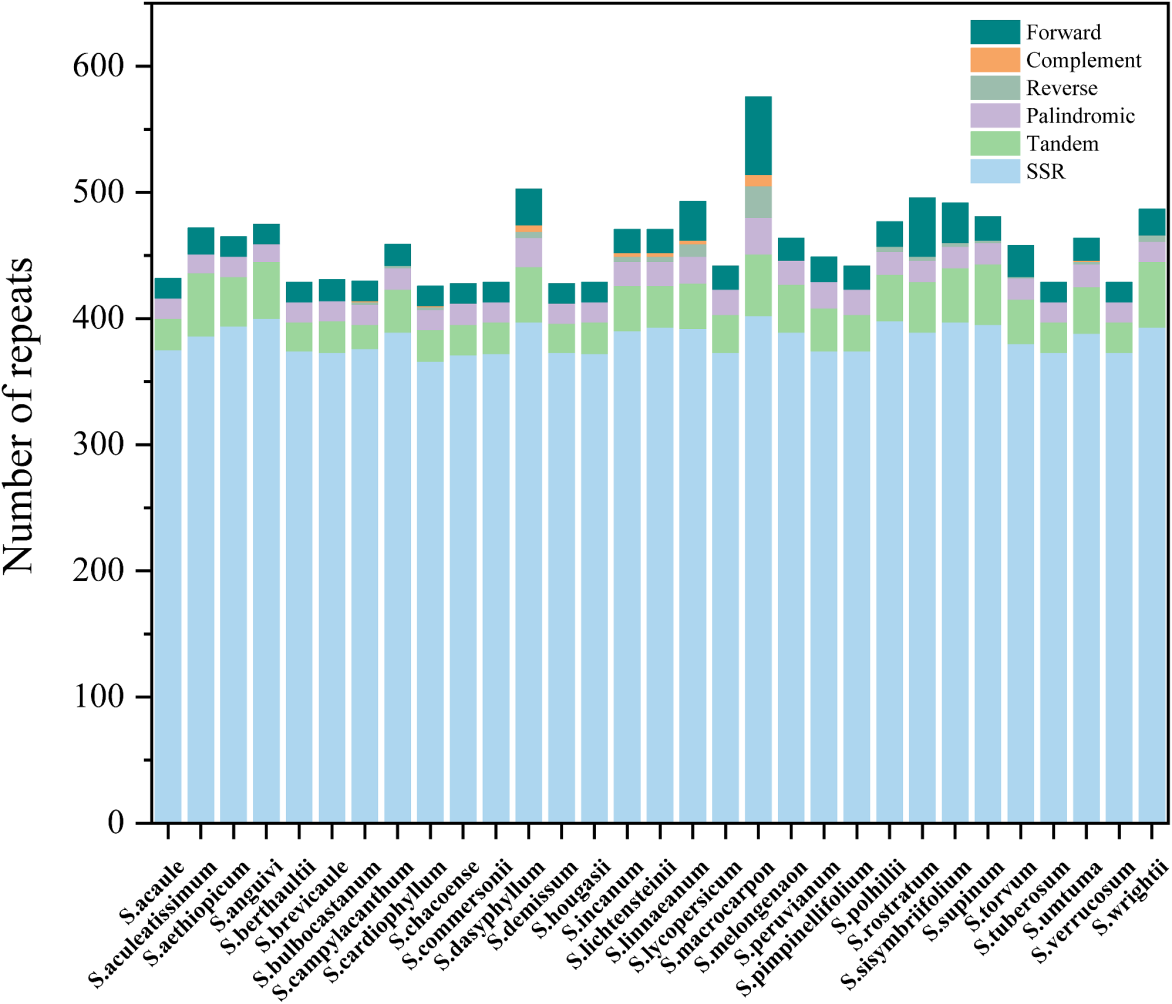
Analyses of repeat sequences in 31 *Solanum* chloroplast genomes

Most of the Large Simple Repeat (LSR) sequences fell within the range of 30–50, with only a few species having LSRs exceeding 60 (Fig. 5 and Table S7). Palindromic and Forward types were the most abundant, while Reverse and Complement types were less common and absent in many species. The number of tandem repeat sequences remained relatively stable, ranging from 24 to 52.

### Identification of the most variable regions

The DnaSP program was employed to conduct a thorough analysis of nucleotide polymorphisms in the 31 chloroplast genomes, with the aim of calculating nucleotide diversity (pi). The analysis revealed a total of 752 polymorphic loci within the chloroplast genome, distributed as follows: 382 in the LSC region, 88 in the SSC region, and 282 in the IR region. When considering diversity values (pi), the highest average value of pi was observed in the SSC region, registering at 0.01199. In contrast, the IR region exhibited the lowest average value of pi at 0.00579 (Table S8).

**Fig. 6:**
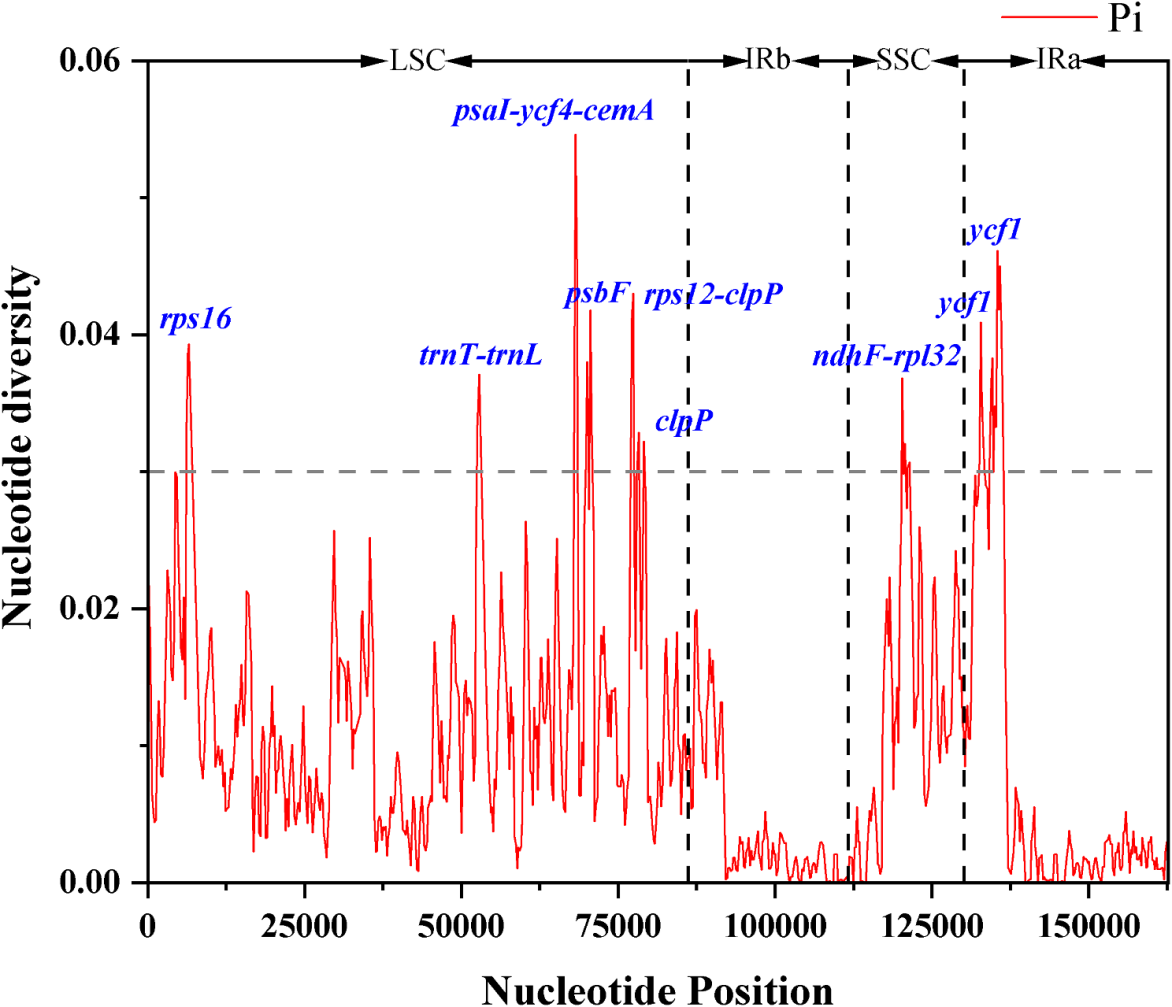
Sliding window analysis of the whole cp. genomes of 31 *Solanum* plants. Window length: 600 bp, step size: 200 bp. X-axis, the position of the midpoint of a window; Y-axis, nucleotide diversity of each window

Furthermore, we considered sites with pi values greater than 0.03 as highly variable sites. Among these, six were located in the LSC region (*rps16*, *trnT-trnL*, *psaI-ycf4-cemA*, *psbF*, *rps12-clpP*, and *clpP*), one in the SSC region (*ndhF-rpl32*), and one was identified in the IR region (*ycf1*) (Fig. 6). Notably, the SSC and LSC regions exhibited higher nucleotide polymorphism compared to the IR region, indicating differentiation in the chloroplast LSC and SSC regions, while the IR region remained relatively conserved. This observation aligns with the results obtained from mVIISTA analysis. The eight highly variable loci identified can serve as valuable candidate molecular markers for the identification of *Solanum* plants.

### Comparison of gene content

**Fig. 7:**
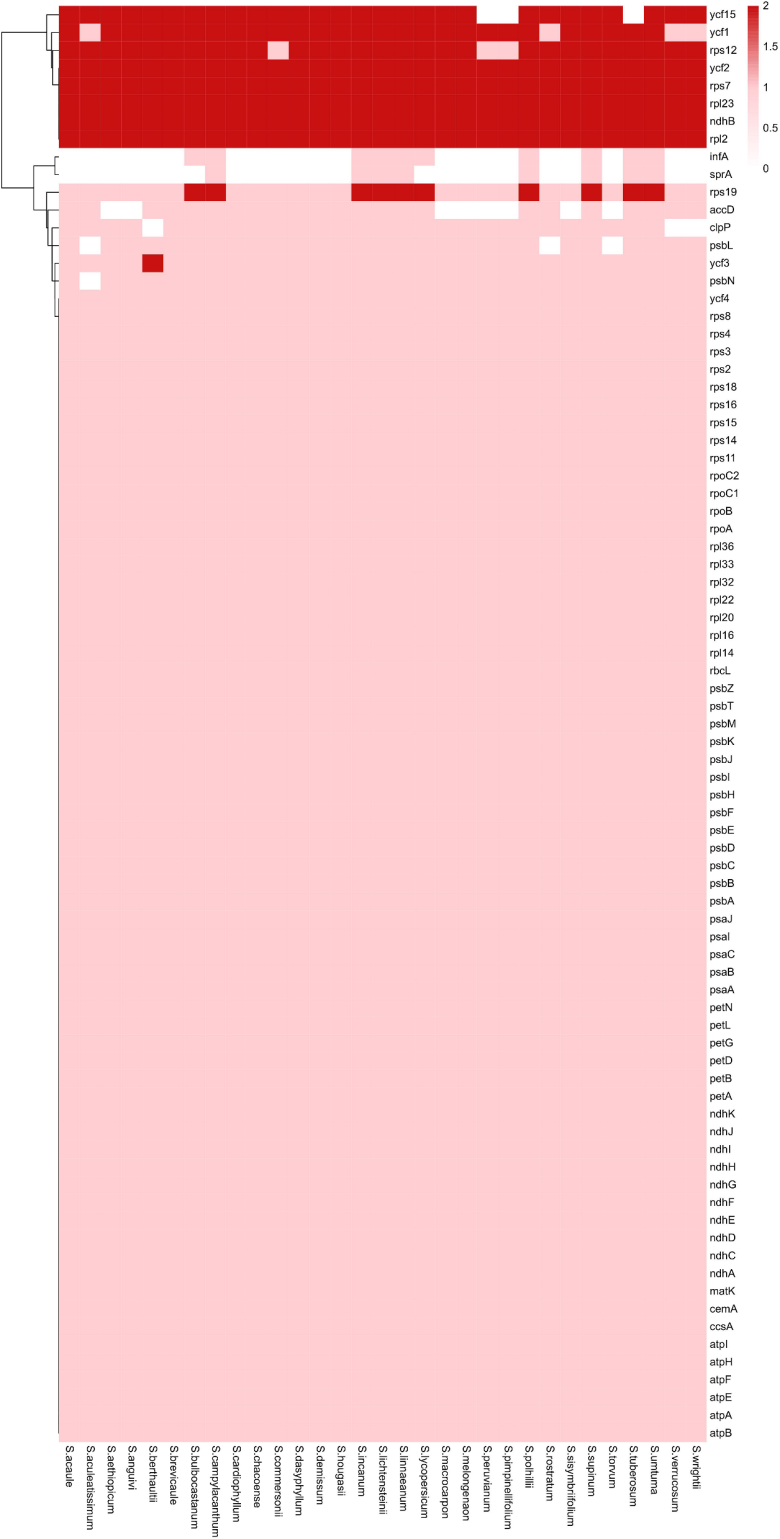
Deletion of chloroplast genes in 31 species of solanum plants

Although chloroplasts are generally conserved during development, they have experienced varying degrees of gene deletions throughout their long evolutionary history. After summarizing the 31 chloroplast genome genes, we identified 11 chloroplast genes that have undergone deletions or additions. Among the more. significant deletions are those of *infA*, *sprA*, and *accD* (Fig. 7).

### Phylogenetic analysis

**Fig. 8:**
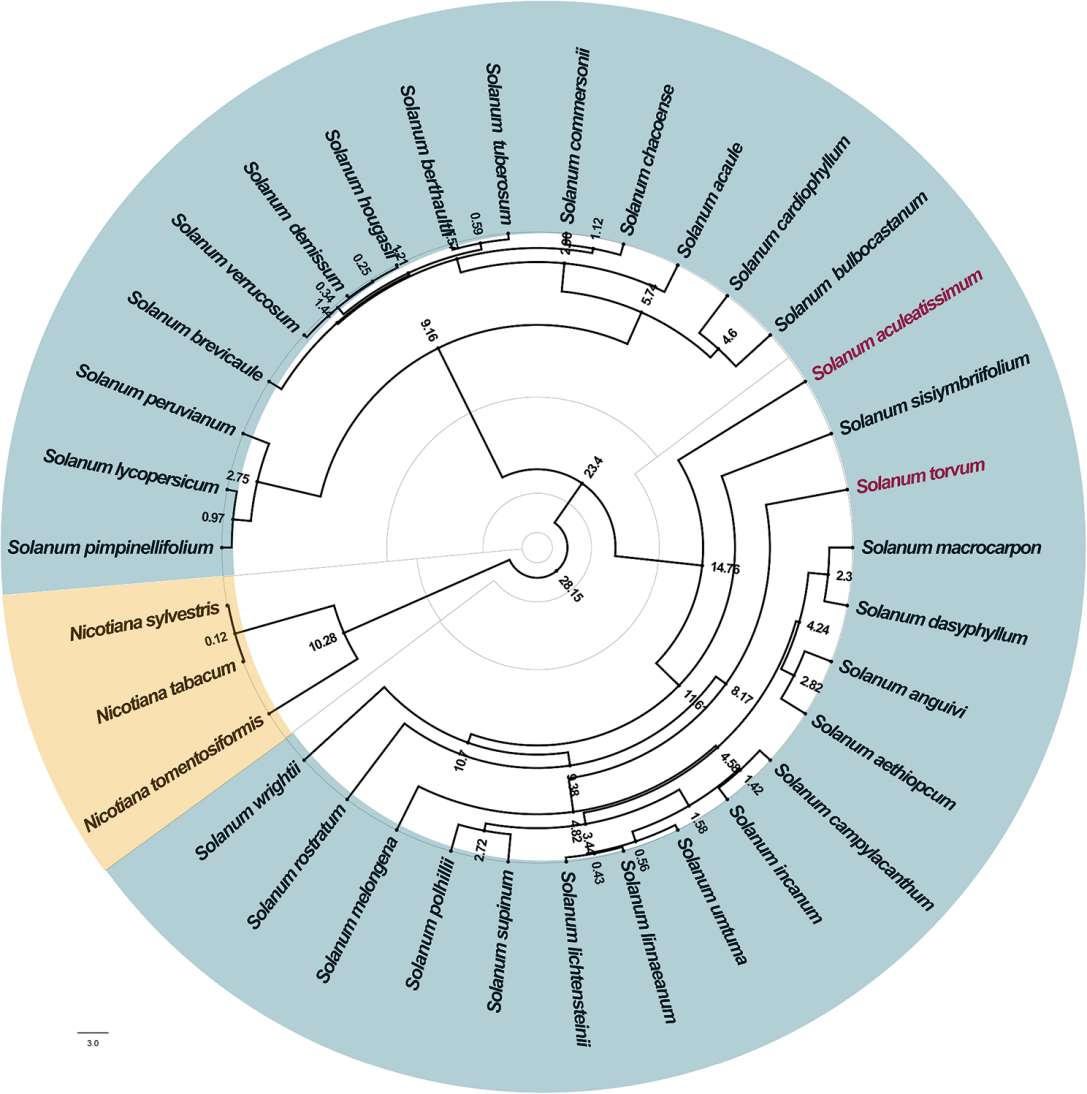
Phylogenetic tree of differentiation times for 34 species of Solanaceae. The numbers near the nodes in the figure indicate the time of divergence of the corresponding species in millions of years (mya)

To investigate the affinities among the 31 *Solanum* species, we constructed a phylogenetic evolutionary tree through comparative analyses of genes shared within chloroplasts. Additionally, we introduced three *Nicotiana* species as outgroups (*Nicotiana sylvestris*, *Nicotiana tabacum*, and *Nicotiana tomentosiformis*). The evolutionary tree revealed the presence of two distinct strong branches outside the outgroups.One branch consistently comprised tomato, potato, and their wild relatives, while the other branch included cultivated eggplant and its wild relatives, with the newly sequenced *S. aculeatissimum* and *S. torvum* positioned within this branch. Notably, *S. aculeatissimum* occupied the outermost position in this branch, whereas *S. torvum* was positioned closer to its center (Fig. 8). This observation underscores that *S. aculeatissimum* is more distantly related to cultivated eggplant compared to *S. torvum*, aligning with previous studies [36, 37] and receiving strong support. From the perspective of divergence time, the earliest divergence occurred in plants of the genus Tobacco approximately 28 million years ago (mya). Subsequently, there was another divergence within the genus Solanum around 23 mya. Over time, more and more species underwent successive divergences. Finally, the majority of species within the *Solanum* genus underwent differentiation approximately 5 to 2.5 million years ago. Among the two species sequenced in this study, *S. aculeatissimum* and *S. torvum* diverged around 14.76 mya and 8.17 mya, respectively.

## Disscusion

In this study, we sequenced, assembled, and annotated the complete chloroplast genomes of two Solanum species, *S. aculeatissimum* and *S. torvum*. We then combined these data with the published chloroplast genome sequences of 29 other species for comparative analysis. The results indicated that the chloroplast genomes of these species did not exhibit significant variations in size, and they were relatively conserved in terms of gene number, structure, and location, displaying minimal variation. This conservation could be attributed to the predominantly maternal inheritance of plastids in the process of angiosperm inheritance [38].

An analysis of GC content in each region revealed that the Inverted Repeat (IR) region had a significantly higher GC content compared to the Large Single Copy (LSC) and Small Single Copy (SSC) regions. This phenomenon can be attributed to the presence of rRNA in the IR region, leading to an elevated GC content [39, 40].

Regarding gene deletions, the occurrence of missing genes in *Solanum* species is relatively rare, with deletions mainly affecting *infA*, *sprA*, and *accD*. The *infA* gene encodes a protein translation initiation factor IF1, approximately 70 amino acids in length, which plays a crucial role in the initiation of protein translation in organellar species [41, 42]. Subsequent studies have revealed that *InfA* exhibits varying levels of activity in the chloroplast genome across evolutionary species, mutating in some and disappearing in others [43]. The *accD* gene undergoes RNA editing and is involved in the regulation of ACCase activity and fatty acid synthesis in response to high-temperature stress. Hence, its absence in some species may indicate its decreasing importance in individual species over the course of evolution. The function of the *sprA* gene in plant chloroplasts remains unclear.

In addition to these findings, In the chloroplast genomes of both *S. aculeatissimum and S. torvum*, there are two *tRNA-UUC* genes. However, notably, each of the two *tRNA-UUC* genes in the chloroplast genome of *S. aculeatissimum* contains an intron, whereas in the chloroplast genome of *S. torvum*, only one of the two *tRNA-UUC* genes harbors an intron. Codon preference analysis revealed that both *S. aculeatissimum* and *S. torvum* exhibited weaker codon preferences, though differences between the two species were still evident. These differences may be attributed to the natural selection processes that different species have undergone throughout their evolutionary history [44, 45]. Furthermore, variations exist in the types of amino acids encoded, with the consistent observation that leucine is the most frequently encoded amino acid, while cysteine is the least common.

Numerous studies have demonstrated that the expansion and contraction of the Inverted Repeat (IR) region are among the primary factors contributing to changes in the size of chloroplast genomes [46–48]. In the case of the 31 Solanum chloroplast genomes analyzed here, we observed evidence of contraction and expansion at the IR boundary, with the most significant changes occurring in the IR and Small Single Copy (SSC) regions. In both of these regions, two genes alternated at the boundary. Although the Inverted Repeat (IR) and Large Single Copy (LSC) regions remained relatively conserved, the genes at this boundary exhibited varying degrees of displacement.

Comparative analysis of genome structure and Identification of the most variable regions,indicating that chloroplast genomic variation in Solanum species primarily occurs in the LSC and SSC regions, consistent with the results of previous studies [49, 50]. Nevertheless, nucleotide polymorphism varies among different species, necessitating the customization of distinct highly variable regions as candidate markers. In this experiment, we screened eight candidate regions across the 31 chloroplast genomes, but further research is required to draw precise conclusions.

Simple Sequence Repeat (SSR), as a classical molecular marker technology, is widely utilized in gene localization and phylogenetic studies owing to its high specificity [51]. In *Solanum* species, approximately 65% of all single nucleotides consist of A/T bases, with only about 8% being CG base pairs, a pattern frequently observed in other studies [52–54]. Furthermore, the abundance of repeat sequences decreases as the length of repeat sequences increases. Among the repeat sequences composed of polynucleotides, the individual bases exhibit a bias towards A and T. Research has indicated that the pressure on GC content increases with the rise in A + T content in high A + T regions, leading to a tendency for CG pairs to be lost in such regions [55].

From the results of phylogenetic analysis, it is evident that the genus *Nicotiana* forms a distinct and robust monophyletic group, diverging earliest and demonstrating a relatively distant relationship with the genus *Solanum*. In future molecular biology studies, valuable genes can be explored within the *Nicotiana* genus and transferred for research within the *Solanum* genus. Following the divergence of the *Nicotiana* genus, the genus *Solanum* further differentiates into two branches. One branch is represented by plants such as tomatoes and potatoes, while the other is represented by plants like eggplants– the two species sequenced in this study belong to this latter branch.

Within these two branches, the relationship is closer compared to the *Nicotiana* genus. Modern biotechnological techniques, such as somatic cell fusion, can be employed for research. For species within each branch, the closer relationship allows for free grafting between many species. Some species can even hybridize naturally in the wild, producing sterile F_1_ hybrids. These species can be used to cultivate new varieties through artificial hybridization breeding, thereby expanding the genetic resources of the *Solanum* genus.

## Conclusions

With the development of sequencing technology in recent years, more and more researchers have started to analyse chloroplast genomes. In this study, we conducted a comparative genome analysis of 31 chloroplasts of *Solanum* species including the present sequencing. The results revealed that chloroplast genomes across these species exhibited a high degree of stability in terms of size, gene content, structure, and location. This conservation can be attributed to the prevalent maternal inheritance pattern in angiosperm reproduction. Additionally, we observed a significantly elevated GC content in the Inverted Repeat (IR) region, primarily due to the presence of rRNA genes.Furthermore, gene deletions were identified in specific *Solanum* species, including *infA*, *sprA*, and *accD*. These deletions likely represent adaptive responses to evolving environmental and physiological requirements.Our comparative analysis highlighted that variations in chloroplast genomes primarily occurred in the Large Single Copy (LSC) and Small Single Copy (SSC) regions, consistent with prior research. We also conducted an analysis of Simple Sequence Repeats (SSRs), revealing a predominance of A/T base pairs, which holds significance for species identification and evolutionary studies. Also on this basis, we introduced three plants of the genus *Nicotiana* in the family *Solanaceae* to construct a phylogenetic evolutionary tree together with 31 species of *Solanum*, and estimated the divergence time of these species. This is rarely seen in chloroplast genome analyses of *Solanaceae*. Finally, the phylogenetic analysis indicated a closer relationship between *S. aculeatissimum* and *S. torvum* with cultivated eggplant, whereas their relationship with potato and tomato relatives is more distant. This finding suggests that interspecific crosses may prioritize species within the same branch as *S. aculeatissimum* and *S. torvum*, offering valuable insights for the adaptive evolution and breeding of *Solanum* species.

### Electronic supplementary material

Below is the link to the electronic supplementary material.

Supplementary Material 1: Additional fle 1: table S1. List of genes annotated in the cp. genomes of *Solanum aculeatissimum* sequenced in this study.

Supplementary Material 2: Additional fle 2: table S2. List of genes annotated in the cp. genomes of *Solanum torvum* sequenced in this study.

Supplementary Material 3: Additional fle 3: table S3. Codon-anticodon recognition patterns and condon usage of the *Solanum aculeatissimum* Chloroplast genome.

Supplementary Material 4: Additional fle 4: table S4. Codon-anticodon recognition patterns and condon usage of the*Solanum torvum* Chloroplast genome.

Supplementary Material 5: Additional fle 5: table S5. Quantity and types of simple repeat sequences(SSR).

Supplementary Material 6: Additional fle 6: table S6. Quantity and types of repeat sequences.

Supplementary Material 7: Additional fle 7: table S7. Average Pi values in different regions of *Solanum* chloroplast genomes.

Supplementary Material 8:Additional fle 8: table S8.Nucleotide polymorphism in different regions.

## Data Availability

The complete chloroplast genome of *Solanum aculatissimum* and *Solanum torvum* have been deposited in the NCBI repository, https://www.ncbi.nlm.nih.gov/nuccore/OR381845.1/ and https://www.ncbi.nlm.nih.gov/nuccore/OR381846.1/. The remaining 29 chloroplast genome data for comparison can be obtained using the registration numbers provided in Table 1 of this article at the GenBank of NCBI.

## Abbreviations

LSC: copy region Large single
SSC: copy region Small single
IR: Inverted repeat region
CDS: Coding DNA sequence
tRNAs: Transport RNAs
rRNAs: Ribosomal RNAs
RSCU: Relative synonymous codon usage
SSR: Simple sequence repeat
LSR: Large sequence repeat
Pi: Nucleotide diversity
NCBI: National Center for Biotechnology Information
*S.acaule*: *Solanum acaule*
*S.aculeatissimum*: *Solanum aculeatissimum*
*S.aethiopicum*: *Solanum aethiopicum*
*S.anguivi*: *Solanum anguivi*
*S.berthaultii*: *Solanum berthaultii*
*S.brevicaule*: *Solanum brevicaule*
*S.bulbocastanum*: *Solanum bulbocastanum*
*S.campylacanthum*: *Solanum campylacanthum*
*S.cardiophyllum*: *Solanum cardiophyllum*
*S.chacoense*: *Solanum chacoense*
*S.commersonii*: *Solanum commersonii*
*S.dasyphyllum*: *Solanum dasyphyllum*
*S.demissum*: *Solanum demissum*
*S.hougasii*: *Solanum hougasii*
*S.incanum*: *Solanum incanum*
*S.lichtensteinii*: *Solanum lichtensteinii*
*S.linnaeanum*: *Solanum linnaeanum*
*S.lycopersicum*: *Solanum lycopersicum*
*S.macrocarpon*: *Solanum macrocarpon*
*S.melongenaon*: *Solanum melongenaon*
*S.peruvianum*: *Solanum peruvianum*
*S.pimpinellifolium*: *Solanum pimpinellifolium*
*S.polhillii*: *Solanum polhillii*
*S.rostratum*: *Solanum rostratum*
*S.sisymbriifolium*: *Solanum sisymbriifolium*
*S.supinum*: *Solanum supinum*
*S.torvum*: *Solanum torvum*
*S.tuberosum*: *Solanum tuberosum*
*S.umtuma*: *Solanum umtuma*
*S.verrucosum*: *Solanum verrucosum*
*S.wrightii*: *Solanum wrightii*
