# Supplementary material for: Solanum aculeatissimum and Solanum torvum chloroplast genome sequences: a comparative analysis with other Solanum chloroplast genomes
Source: BMC Genomics. 2024 Apr 26;25:412. doi: 10.1186/s12864-024-10190-9 (PMC11046870; doi:10.1186/s12864-024-10190-9)
Supplement: Supplementary file 1 — Supplementary Material 1: Additional fle 1: table S1. List of genes annotated in the cp. genomes of Solanum aculeatissimum sequenced in this study. [file 12864_2024_10190_MOESM1_ESM.docx]

Table S1 Assessment statistics of sequencing data in Solanum aculeatissimum、Solanum torvum

| Samples | Read Number | Base Number | GC Content | %≥Q30 | Cholroplast genome coverage(×) |
| --- | --- | --- | --- | --- | --- |
| *Solanum aculeatissimum* | 23961469 | 7188440700 | 37.10 | 91.11 | 200 |
| *Solanum torvum* | 22562642 | 6768792600 | 37.30 | 91.51 | 190 |

Note: Samples: Sample name of sample information sheet；ReadSum: Total pair-end Reads in Clean Data；BaseSum: Total base number of Clean Data;GC content: Clean Data GC content, the percentage of G and C bases in Clean Data to total bases；%≥Q30：The percentage of bases whose mass value of Clean Data is greater than or equal to 30.
