# Supplementary material for: Solanum aculeatissimum and Solanum torvum chloroplast genome sequences: a comparative analysis with other Solanum chloroplast genomes
Source: BMC Genomics. 2024 Apr 26;25:412. doi: 10.1186/s12864-024-10190-9 (PMC11046870; doi:10.1186/s12864-024-10190-9)
Supplement: Supplementary file 2 — Supplementary Material 2: Additional fle 2: table S2. List of genes annotated in the cp. genomes of Solanum torvum sequenced in this study. [file 12864_2024_10190_MOESM2_ESM.docx]

**Table S2:** List of genes annotated in the cp genomes of *Solanum aculeatissimum* sequenced in this study.

|  | Category | Name of Gene | | | |
| --- | --- | --- | --- | --- | --- |
| Self-replication | Ribosomal RNA | *rrn16* | *rrn23* | *rrn4.5* | *rrn5* |
|  | Transfer RNA | *tRNA-GUG* | *tRNA-UUU^a^* | *tRNA-UUG* | *tRNA-GCU* |
|  |  | *tRNA-CGA^a^* | *tRNA-UCU* | *tRNA-GCA* | *tRNA-GUC* |
|  |  | *tRNA-GUA* | *tRNA-UUC* | *tRNA-GGU* | *tRNA-UGA* |
|  |  | *tRNA-GCC* | *tRNA-CAU* | *tRNA-GGA* | *tRNA-UGU* |
|  |  | *tRNA-UAA^a^* | *tRNA-GAA* | *tRNA-UAC^a^* | *tRNA-CAU* |
|  |  | *tRNA-CCA* | *tRNA-UGG* | *tRNA-CAU* | *tRNA-CAA* |
|  |  | *tRNA-GAC* | *tRNA-UUC^a^* | *tRNA-UGC* | *tRNA-ACG* |
|  |  | *tRNA-GUU* | *tRNA-UAG* | *tRNA-GUU* | *tRNA-ACG* |
|  |  | *tRNA-UGC^a^* | *tRNA-CAU* | *tRNA-GAC* | *tRNA-CAA* |
|  |  |  |  |  |  |
|  | Small subunit of ribosome | *rps2* | *rps14* | *rps4* | *rps18* |
|  |  | *rps12^a^* | *rps11* | *rps8* | *rps3* |
|  |  | *rps19* | *rps7* | *rps15* | *rps7* |
|  | Large subunit of ribosome | *rpl33* | *rpl20* | *rpl36* | *rpl14* |
|  |  | *rpl16* | *rpl22* | *rpl2* | *rpl23* |
|  |  | *rpl32* |  |  |  |
|  | RNA polymerase subunits | *rpoC2* | *rpoC1^a^* | *rpoB* | *rpoA* |
| photosynthesis | Subunits of photosynthesisⅠ | *psaB* | *psaA* | *psaI* | *psaJ* |
|  |  | *psaC* |  |  |  |
|  | Subunits of photosynthesisⅡ | *psbA* | *psbK* | *psbI* | *psbM* |
|  |  | *psbD* | *psbC* | *psbZ* | *psbJ* |
|  |  | *psbF* | *psbE* | *psbB* | *psbT* |
|  |  | *psbN* | *psbH* |  |  |
|  | Subunits of cytochrome | *petN* | *petA* | *petL* | *petG* |
|  |  | *petB* | *petD* |  |  |
|  | Subunits of ATP synthase | *atpA* | *atpF* | *atpH* | *atpI* |
|  |  | *atpE* | *atpB* |  |  |
|  | Large subunit of RuBisCo | *rbcL* |  |  |  |
|  | Subunits of NADH | *ndhJ* | *ndhK* | *ndhC* | *ndhB^a^* |
|  |  | *ndhF* | *ndhE* | *ndhG* | *ndhI* |
|  |  | *ndhA^a^* | *ndhH* | *ndhD* |  |
| Other gene | Maturase | *matK* |  |  |  |
|  | Envelope membrane protein | *cemA* |  |  |  |
|  | Subunit of acetyl-CoA | *accD* |  |  |  |
|  | C-type cytochrome synthesis gene | *ccsA* |  |  |  |
|  | Protease | *clpP^a^* |  |  |  |
| Unknown function | Conserved open reading frames | *ycf3* | *ycf4* | *ycf2* | *ycf15* |
|  |  | *ycf1* |  |  |  |

Note:*a* : gene containing an intron
