# Supplementary material for: Solanum aculeatissimum and Solanum torvum chloroplast genome sequences: a comparative analysis with other Solanum chloroplast genomes
Source: BMC Genomics. 2024 Apr 26;25:412. doi: 10.1186/s12864-024-10190-9 (PMC11046870; doi:10.1186/s12864-024-10190-9)
Supplement: Supplementary file 4 — Supplementary Material 4: Additional fle 4: table S4. Codon-anticodon recognition patterns and condon usage of theSolanum torvum Chloroplast genome. [file 12864_2024_10190_MOESM4_ESM.docx]

Table S4. Codon-anticodon recognition patterns and condon usage of the *Solanum aculeatissimum* Chloroplast genome.

| Amino Acid | Codon | No. | RESCU | o Acid | Codon | No. | RESCU |
| --- | --- | --- | --- | --- | --- | --- | --- |
| Ala | GCA | 401 | 1.16 | Leu | CUA | 388 | 0.83 |
|  | GCC | 231 | 0.67 |  | CUC | 189 | 0.41 |
|  | GCG | 136 | 0.39 |  | CUG | 184 | 0.40 |
|  | GCU | 615 | 1.78 |  | CUU | 613 | 1.32 |
| Cys | UGC | 77 | 0.51 |  | UUA | 851 | 1.83 |
|  | UGU | 224 | 1.49 |  | UUG | 567 | 1.22 |
| Asp | GAC | 255 | 0.42 | Met | AUG | 613 | 1.99 |
|  | GAU | 843 | 1.58 |  | GUG | 3 | 0.01 |
| Glu | GAA | 1007 | 1.49 | Asn | AAC | 310 | 0.48 |
|  | GAG | 347 | 0.51 |  | AAU | 972 | 1.52 |
| Phe | UUC | 525 | 0.71 | Pro | CCA | 331 | 1.19 |
|  | UUU | 961 | 1.29 |  | CCC | 204 | 0.74 |
| Gly | GGA | 709 | 1.58 |  | CCG | 160 | 0.58 |
|  | GGC | 200 | 0.45 |  | CCU | 415 | 1.50 |
|  | GGG | 324 | 0.72 | Gln | CAA | 713 | 1.52 |
|  | GGU | 561 | 1.25 |  | CAG | 288 | 0.48 |
| His | CAC | 138 | 0.45 | Arg | AGA | 469 | 1.77 |
|  | CAU | 469 | 1.55 |  | AGG | 168 | 0.63 |
| Ile | AUA | 665 | 0.91 |  | CGA | 381 | 1.44 |
|  | AUC | 457 | 0.62 |  | CGC | 104 | 0.40 |
|  | AUU | 1078 | 1.47 |  | CGG | 120 | 0.45 |
| Lys | AAA | 1031 | 1.47 |  | CGU | 346 | 1.31 |
|  | AAG | 376 | 0.53 | Val | GUA | 519 | 1.47 |
| Ser | AGC | 127 | 0.37 |  | GUC | 183 | 0.52 |
|  | AGU | 391 | 1.15 |  | GUG | 195 | 0.55 |
|  | UCA | 408 | 1.20 |  | GUU | 519 | 1.47 |
|  | UCC | 330 | 0.97 | Trp | UGG | 479 | 1.00 |
|  | UCG | 206 | 0.61 | Tyr | UAC | 186 | 0.39 |
|  | UCU | 579 | 1.70 |  | UAU | 771 | 1.61 |
| Thr | ACA | 407 | 1.22 |  |  |  |  |
|  | ACC | 258 | 0.77 |  |  |  |  |
|  | ACG | 145 | 0.43 |  |  |  |  |
|  | ACU | 525 | 1.57 |  |  |  |  |
