# Supplementary material for: Solanum aculeatissimum and Solanum torvum chloroplast genome sequences: a comparative analysis with other Solanum chloroplast genomes
Source: BMC Genomics. 2024 Apr 26;25:412. doi: 10.1186/s12864-024-10190-9 (PMC11046870; doi:10.1186/s12864-024-10190-9)
Supplement: Supplementary file 5 — Supplementary Material 5: Additional fle 5: table S5. Quantity and types of simple repeat sequences(SSR). [file 12864_2024_10190_MOESM5_ESM.docx]

Table S5. Codon-anticodon recognition patterns and condon usage of the*Solanum torvum* Chloroplast genome.

| Amino Acid | Codon | No. | RESCU | Amino Acid | Codon | No. | RESCU |
| --- | --- | --- | --- | --- | --- | --- | --- |
| Ala | GCA | 402 | 1.15 | Leu | CUA | 389 | 0.83 |
|  | GCC | 235 | 0.67 |  | CUC | 196 | 0.42 |
|  | GCG | 136 | 0.39 |  | CUG | 192 | 0.41 |
|  | GCU | 622 | 1.78 |  | CUU | 614 | 1.30 |
| Cys | UGC | 78 | 0.51 |  | UUA | 858 | 1.82 |
|  | UGU | 221 | 1.48 |  | UUG | 578 | 1.23 |
| Asp | GAC | 221 | 0.41 | Tyr | UAC | 184 | 0.38 |
|  | GAU | 846 | 1.59 |  | UAU | 773 | 1.62 |
| Glu | GAA | 1015 | 1.48 | Asn | AAC | 309 | 0.48 |
|  | GAG | 355 | 0.52 |  | AAU | 972 | 1.52 |
| Phe | UUC | 527 | 0.70 | Pro | CCA | 326 | 1.18 |
|  | UUU | 971 | 1.30 |  | CCC | 206 | 0.75 |
| Gly | GGA | 719 | 1.59 |  | CCG | 154 | 0.56 |
|  | GGC | 195 | 0.43 |  | CCU | 419 | 1.52 |
|  | GGG | 324 | 0.72 | Gln | CAA | 705 | 1.49 |
|  | GGU | 568 | 1.26 |  | CAG | 239 | 0.51 |
| His | CAC | 140 | 0.45 | Arg | AGA | 471 | 1.77 |
|  | CAU | 477 | 1.55 |  | AGG | 173 | 0.65 |
| Ile | AUA | 666 | 0.90 |  | CGA | 385 | 1.44 |
|  | AUC | 451 | 0.61 |  | CGC | 106 | 0.40 |
|  | AUU | 1102 | 1.49 |  | CGG | 118 | 0.44 |
| Lys | AAA | 1049 | 1.48 |  | CGU | 348 | 1.30 |
|  | AAG | 369 | 0.52 | Val | GUA | 524 | 1.47 |
| Ser | AGC | 123 | 0.36 |  | GUC | 185 | 0.52 |
|  | AGU | 403 | 1.17 |  | GUG | 196 | 0.55 |
|  | UCA | 414 | 1.21 |  | GUU | 519 | 1.46 |
|  | UCC | 338 | 0.99 | Trp | UGG | 472 | 1.00 |
|  | UCG | 199 | 0.58 | Thr | ACA | 408 | 1.22 |
|  | UCU | 581 | 1.69 |  | ACC | 257 | 0.77 |
| Met | AUA | 1 | 0.01 |  | ACG | 148 | 0.44 |
|  | AUG | 615 | 3.97 |  | ACU | 525 | 1.57 |
|  | GUG | 3 | 0.02 |  |  |  |  |
|  | UUG | 1 | 0.01 |  |  |  |  |
